# Urine cell-free microRNA as biomarkers for transitional cell carcinoma

**DOI:** 10.1186/s13104-017-2950-9

**Published:** 2017-11-29

**Authors:** Gil A. Geva, Ilan Gielchinsky, Nina Aviv, Klaas E. A. Max, Ofer N. Gofrit, Devorah Gur-Wahnon, Iddo Z. Ben-Dov

**Affiliations:** 1 Nephrology and Hypertension, Hadassah-Hebrew University Medical Center, POB 12000, 91120 Jerusalem, Israel; 2 Department of Urology, Hadassah-Hebrew University Medical Center, POB 12000, 91120 Jerusalem, Israel; 3 Laboratory of RNA Molecular Biology, The Rockefeller University, New York, NY 10065 USA

**Keywords:** Transitional cell carcinoma, Urothelial carcinoma, Biomarkers, microRNA, Extracellular RNA

## Abstract

**Objective:**

MicroRNA (miRNA) are short nucleotide strands with a regulatory function in the cell. Several miRNAs have been shown to be useful as biomarkers for different neoplasms. The aim of this project was to assess whether levels of miRNA in cell free urine could be used as a biomarker in transitional cell carcinoma (TCC).

**Results:**

cDNA libraries were produced based on small RNAs in urine samples of fourteen TCC patients and twenty healthy volunteers. Resulting reads were deep sequenced on Illumina HiSeq sequencer with the intent of characterizing cell free urine miRNA profiles. A statistically significant difference was found for a single miRNA; miR-210 was > sixfold higher in the TCC group compared to the control group. Furthermore, we were able to produce a diagnostic score by summing of standardized levels of overexpressed miRNA. This score was considerably higher in TCC patients with a sensitivity of 0.93, specificity of 0.76 and negative predictive value > 0.97.

**Electronic supplementary material:**

The online version of this article (10.1186/s13104-017-2950-9) contains supplementary material, which is available to authorized users.

## Introduction

Transitional cell carcinoma (TCC) is a histological classification of tumors in the urinary tract. It is the most common neoplasm in the urinary tract and is most frequently found in the urinary bladder. TCC also appears in the renal pelvis, ureter and urethra. It is more prevalent in men, smokers, and with advanced age. It is estimated that 75,000 people are diagnosed with TCC and 16,000 die of the disease every year in the United States alone [1]. The gold standard both for diagnosis and for follow up for recurrence of the disease is cystoscopy: an expensive, invasive and uncomfortable test. Other modalities include fluorescence cystoscopy (cystoscopy in which the photoactive nature of certain compounds is used in order to enhance the visual difference between normal and neoplastic tissue), which has good sensitivity but is not specific; and cytological evaluation of urine, which has low sensitivity and is operator dependent.

The European Association of Urology recommends follow up with cystoscopy 3 months after therapy and then annually for 5 years [2]. As stated earlier this test is expensive, invasive and uncomfortable, but the alternative tests are not reliable enough. This project’s goal is to examine extracellular urine microRNA (miRNA) as a noninvasive, easily obtainable marker for the follow up of TCC patients.

miRNAs are regulatory molecules involved in numerous biological functions. They are single-stranded highly conserved small (20–24 nucleotides long) non-coding RNAs that recognize complementary sequences in the 3′ untranslated regions of target mRNAs, leading to suppression of protein expression by mRNA degradation or by translational repression. It is estimated that 30–80% of human protein coding genes are regulated by several hundred miRNAs [3]. As miRNA are frequently involved in processes of cell death and renewal, miRNA deregulation is a common feature of human malignancies. miRNA have been shown to take part in cellular response to hypoxia and angiogenesis, promote epithelial mesenchymal transition, and affect DNA repair processes [4–6]. Differential expression of miRNA in different tissues and the fact that miRNA are stable in biological fluids lead researchers to believe that miRNA could be used as biomarkers for neoplasms [7, 8]. Indeed miRNA were shown to be a prognostic biomarker in chronic lymphocytic leukemia, lung cancers, hepatocellular carcinomas, melanoma and were found to be differently expressed in colon, thyroid, pancreas, ovarian and cervical carcinomas [9–12]. In this study, we collected urine samples from patients with TCC and from healthy volunteers. We sought out to assess the viability of cell free miRNA in urine as biomarkers for TCC. To that end we extracted and sequenced cell-free miRNA from the urine samples searching for differential expression.

## Main text

### Methods

#### Ethics statement

This study was approved by the Helsinki Committee of the Hadassah Medical Center and the Hebrew University, Jerusalem, Israel. Samples were taken from a different study, previously approved by the ethics board. All clinical specimens were collected after obtaining participants’ written consent.

#### Clinical samples

Urine samples were collected from patients with TCC and from healthy individuals who volunteered specimens to a parallel biomarker study [13]. Patients were excluded from the TCC group if urothelial carcinoma was not confirmed by pathology. As healthy volunteers did not undergo cystoscopies for ethical reasons, control group were generally younger (Additional file 1) to reduce the likelihood of undiagnosed urological tumors. The control group was composed of 20 volunteers with no known urologic diseases; the TCC group initially comprised 18 patients. Four patients were thought to have TCC but were later diagnosed with non-cancer urinary tract disease. These (“other”) patients were not included in the main statistical analysis. Thus, the final TCC group was comprised of 14 patients with proven urothelial carcinoma. Intact cells were separated by centrifugation at 200*g* at 5 °C for 5 min. Cell-free urine samples were frozen at − 80 °C immediately thereafter. Samples, 0.5 ml in volume, were then eluded from the tubes.

#### Isolation of total RNA

Urine samples were incubated with 20 mg/ml proteinase K for 3 min. Organic extraction was then preformed to remove hydrophobic peptide fragments, using a homemade reagent containing 5.91 g guanidinium isothiocyanate (GITC), 25 ml phenol (saturated with 0.1 M citrate buffer pH 4.3), 260 µl β-mercaptoethanol and 3.5 ml Buffer DC (comprised of citric acid, NaOH, Sarcosyl and double distilled water). Following chloroform extraction, isopropanol was added to the aqueous phase. To purify the RNA from the aqueous-isopropanol mixture we used commercial RNeasy MinElute Cleanup columns (Qiagen). The samples then underwent repeated washes under vacuum, followed by elution with double distilled water. Quantifying the RNA was done with the Qubit 2.0 Fluorometer (Thermo Fisher Scientific).

#### Sequencing and annotation of small RNA cDNA libraries

The extracted small RNA underwent barcoded adapter ligation and reverse transcription using Superscript III (Thermo Fisher Scientific). The resulting libraries were deep sequenced on Illumina HiSeq sequencer. The sequence files obtained were separated using the identified barcode sequences. Reads were assigned annotation by comparing to genome and small RNA databases.

#### Statistical analysis

Statistical analysis was done using the DESeq 2 [14] and SAMSeq [15] Bioconductor packages for the R open source software. miRNA were aggregated together into clusters, 1–43 mature miRNA each, based on cistronic location in the genome, in order to reduce error and ease visualizing of the data [16]. To overcome the difficulty of multiple testing the DESeq2 package uses an adjusted *P* value for which we determined that less than 0.05 would be regarded as significant. The SAMseq package utilizes a Q-value to address multiple testing, which we also determined to be significant if < 0.05.

### Results

Cell-free miRNA was collected, sequenced and annotated from 38 urine samples. As mentioned the control group was comprised predominantly of male participants and was younger relative to the TCC group. Additional file 1 depicts the demographic data of the study participants, according to group.

miRNAs were profiled in study participants’ cell-free urine specimens. The 10 most abundant miRNA clusters are shown in Additional file 2; these abundant miRNAs were not differently expressed in patients vs. healthy volunteers (Additional file 3).

**Fig. 1:**
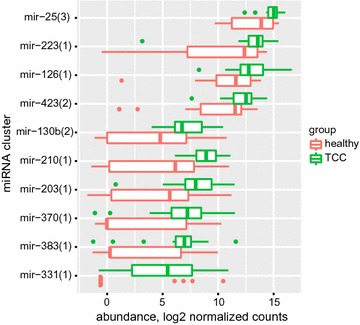
Levels of the upregulated miRNA clusters in the TCC patients’ samples vs. healthy volunteers’ samples, expressed as base-2 logarithm of the normalized counts (box plots)

Differential expression analysis using SAMseq, displayed in Additional files 4 and 5 and in Fig. 1, disclosed 10 less abundant miRNA clusters that were significantly or marginally higher in the patient group compared to controls.

The SAMseq analysis found miR-210 levels to be 6.8 times higher in the TCC cohort compared to the healthy control group, Q value = 0.0 (Additional files 4, 5). This was in line with a DESeq 2 analysis, Additional file 5, where the fold-change was 2.69 (p value was 0.024; adjusted p value 0.433). After adjustment for multiple testing, neither the DESeq 2 method nor the SAMseq method found statistically significant differences in expressions of any other miRNA cluster between the TCC group and the healthy control group.

**Fig. 2:**
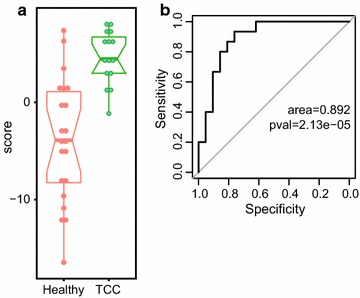
Upregulated miRNA-based diagnostic score values are higher in the TCC vs. healthy subjects (**a** box plots), and perform well in discrimination of patients from controls (**b** receiver operating characteristics (ROC) curve)

Nonetheless, we composed a diagnostic score by summing standardized levels of overexpressed miRNA (Additional file 4). This score was considerably higher in TCC patients compared to healthy controls, Fig. 2a, and had good discrimination ability, as conveyed by the receiver operating characteristics (ROC) curve, Fig. 2b. The sensitivity and specificity values of the miRNA-derived score, at three optimal cut-off points (“OptimalCutpoints” R package, using the minimization of misclassification criterion), are shown in Table 1.

**Table 1:** Diagnostic performance of the upregulated miRNA-based score

Score cut-off	Sensitivity	Specificity	Assumed prevalence 5%		Assumed prevalence 20%	
			PPV	NPV	PPV	NPV
1.140^a^	0.933	0.762	0.171	0.995	0.495	0.979
1.387	0.867	0.810	0.193	0.991	0.532	0.960
2.666	0.800	0.857	0.228	0.988	0.583	0.945

*PPV* positive predictive value, *NPV* negative predictive value

^a^1.140 is the optimal cut-off point according to Youden’s criterion

## Discussion

The European Association of Urology recommends that TCC patients be followed up by cystoscopies post-surgery at least every 6 months for 5 years [2]. Cystoscopies are invasive, expensive and uncomfortable for the patient. They also carry a risk of urinary tract infection, urethral strictures and perforation of the bladder. Cytological examination of urine requires an experienced cytologist and is therefore operator dependent. Use of RT-PCR for miRNA could replace cytology as a more objective, noninvasive follow up test for recurrence of TCC.

The urine miRNA-based diagnostic score depicted in this study provides a good discrimination ability with optimal sensitivity of 0.933 and a negative predicative value of over 0.97. This finding might have translational value as a screening tool for high-risk population; patients with previous occurrence of TCC in which recurrence is common. This score could be used in these patients to accurately rule out the presence of TCC. Additional larger trials are required to validate this finding.

The SAMseq analysis method calculated a Q-value for miR-210 of 0.0 implying that the expression of miR-210 is different (6.8-fold change) between patients with TCC and healthy control groups. miR-210 is largely involved in cellular response to hypoxia. Given that all solid tumors have hypoxic sites, hypoxia response plays an important role in the neoplastic process. miR-210 is regulated by hypoxia inducible factors (HIFs). miR-210 was demonstrated to be elevated in many types of cancer including glioblastoma, melanoma, breast, lung and pancreas [17]. While this result is statistically significant, it is worth mentioning that the DESeq2 method calculated an adjusted P-value of 0.433 for miR-210. Further studies are required in order to understand whether miR-210 is indeed expressed differently.

### Conclusion

We profiled miRNA obtained from cell-free urine samples of both a healthy control cohort and patients with TCC. miR-210 was found to be expressed more in the TCC group compared to the healthy control group. We also produced a molecular score with sensitivity of 93% and a negative predictive value of over 97%. Further research is required to answer for the shortcomings of this study and to validate our findings in larger groups.

## Limitations

We propose several explanations for the low level of statistical significance in our findings. One explanation could be that our sample sizes were simply too small to account for the variance in distribution of cell-free miRNA in urine. If this explanation is true then individual cell-free miRNA in urine are not fit as a screening tool for TCC as they lack sensitivity.

Another explanation could be derived from the difference between the sample cohorts. The TCC group were older with a mean age of 69. The healthy group had a mean age of 37. Both groups were predominantly male. The effect of age on the expression of cell-free miRNA is unknown and may be a confounder.

A third explanation would relate to the fact that we did not stratify the TCC cohort by stage of the disease. It is possible that differences in expression of miRNA only occur in later stages of the disease and by combining samples from different stages we decreased this difference.

## Additional files

**Additional file 1.** Demographic characteristics of study participants.

**Additional file 2.** Levels of the 10 top abundant miRNA clusters in study samples, expressed as base-2 logarithm of the normalized counts.

**Additional file 3.** Levels of the 10 top abundant miRNA clusters in study samples, expressed as base-2 logarithm of the normalized counts, according to study group.

**Additional file 4.** SAMseq analysis of sequencing cDNA libraries—top differentially expressed miRNA clusters in TCC patients vs. control individuals

**Additional file 5.** Differentially expressed miRNA in urine of TCC patients vs. healthy controls by SAMseq and DESeq 2 analyses.

## Abbreviations

TCC: transitional cell carcinoma
miRNA: microRNA
PPV: positive predictive value
NPV: negative predictive value
ROC: receiver operating characteristics
