# Supplementary material for: Urine cell-free microRNA as biomarkers for transitional cell carcinoma
Source: BMC Res Notes. 2017 Nov 29;10:641. doi: 10.1186/s13104-017-2950-9 (PMC5708087; doi:10.1186/s13104-017-2950-9)
Supplement: Supplementary file 1 — Additional file 1. Demographic characteristics of study participants. [file 13104_2017_2950_MOESM1_ESM.docx]

| **Table:** Clinical characteristics of study participants | | | |
| --- | --- | --- | --- |
|  | Healthy, n=20 | TCC, n=14 | Other, n=4 |
| Age (years) | 38.4 | 67.9 | 63.3 |
| Sex (male) | 95% | 78.6% | 100% |
