# Supplementary figures and images for: Urine cell-free microRNA as biomarkers for transitional cell carcinoma
Source: BMC Res Notes. 2017 Nov 29;10:641. doi: 10.1186/s13104-017-2950-9 (PMC5708087; doi:10.1186/s13104-017-2950-9)

miRNA cluster

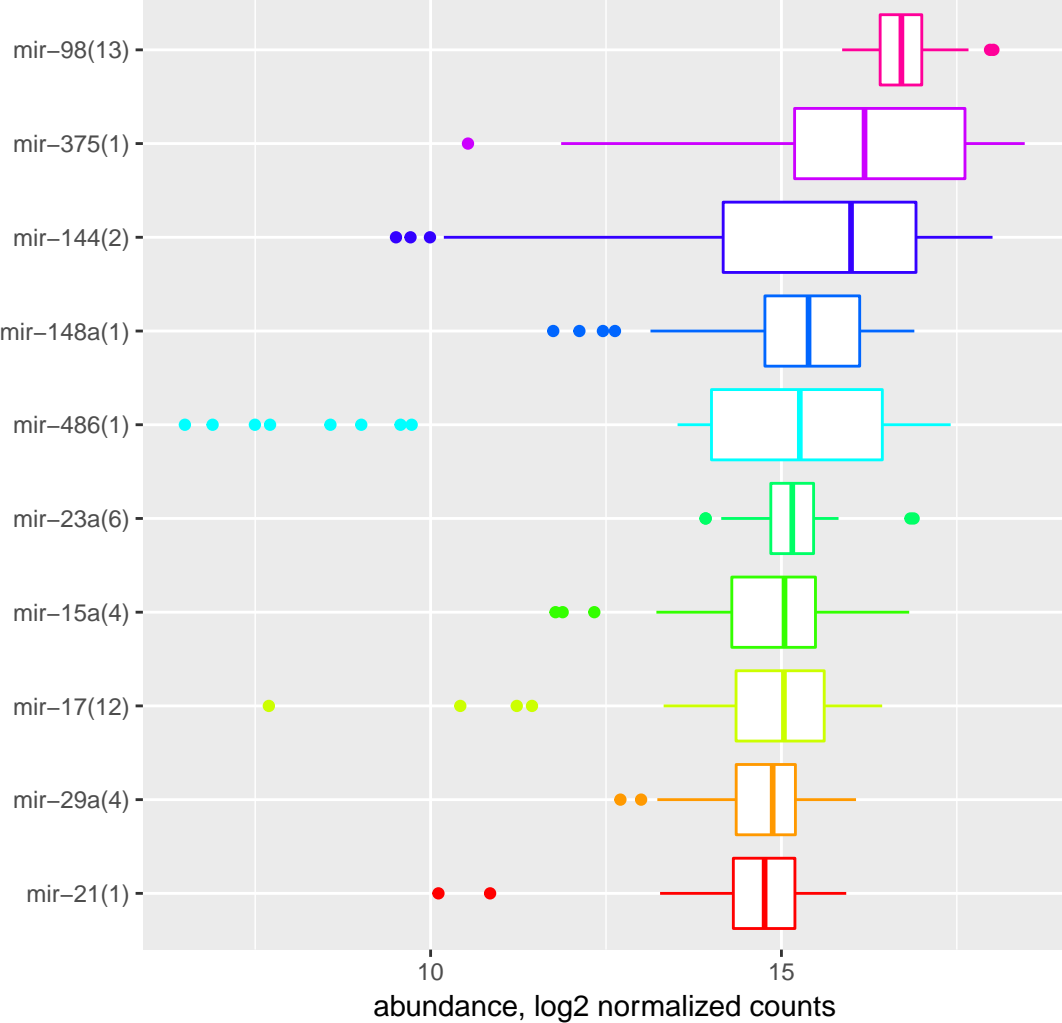

Supplement: Supplementary file 2 — Additional file 2. Levels of the 10 top abundant miRNA clusters in study samples, expressed as base-2 logarithm of the normalized counts. [file 13104_2017_2950_MOESM2_ESM.pdf]

miRNA cluster

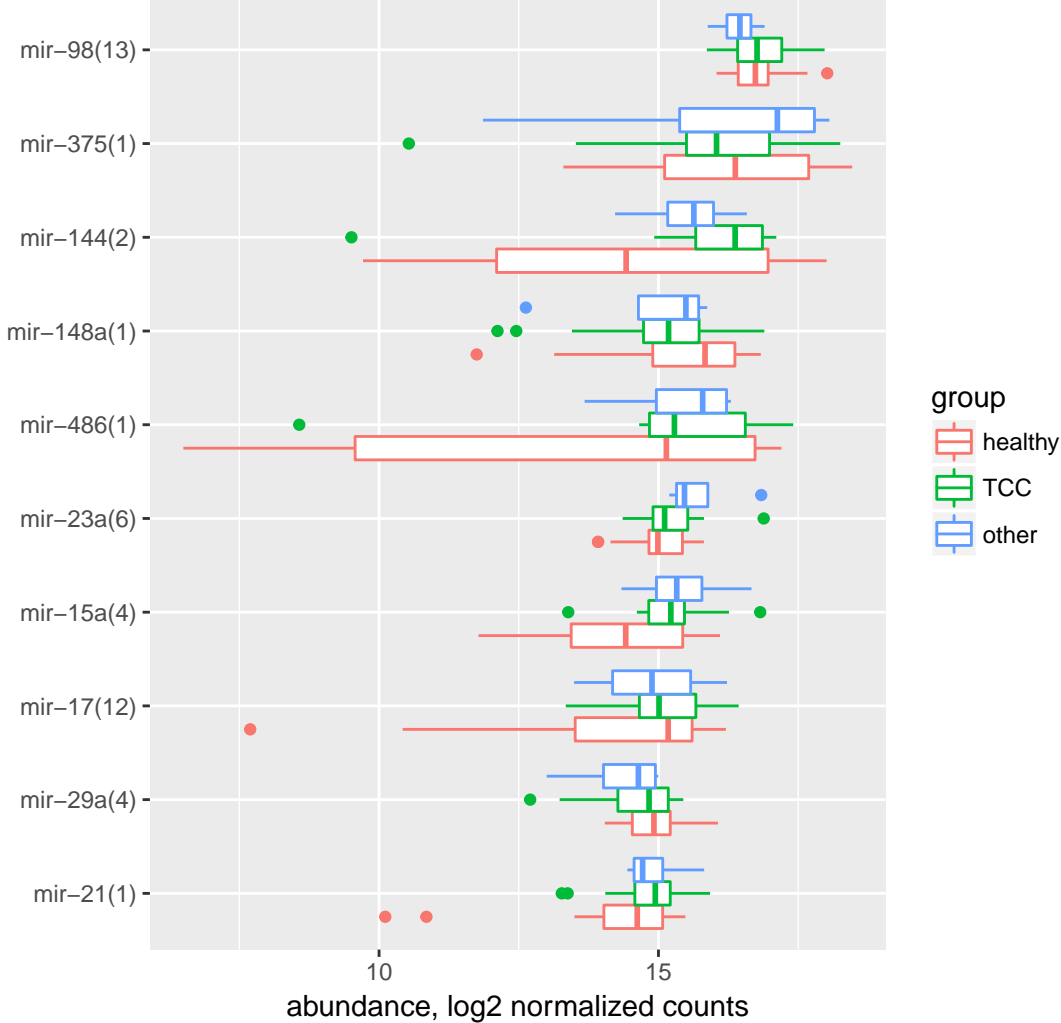

Supplement: Supplementary file 3 — Additional file 3. Levels of the 10 top abundant miRNA clusters in study samples, expressed as base-2 logarithm of the normalized counts, according to study group. [file 13104_2017_2950_MOESM3_ESM.pdf]
