# Supplementary material for: Urine cell-free microRNA as biomarkers for transitional cell carcinoma
Source: BMC Res Notes. 2017 Nov 29;10:641. doi: 10.1186/s13104-017-2950-9 (PMC5708087; doi:10.1186/s13104-017-2950-9)
Supplement: Supplementary file 4 — Additional file 4. SAMseq analysis of sequencing cDNA libraries—top differentially expressed miRNA clusters in TCC patients vs. control individuals [file 13104_2017_2950_MOESM4_ESM.docx]

| **Table:** SAMseq analysis of sequencing cDNA libraries - top differentially expressed miRNA clusters in TCC patients vs. control individuals | | | | |
| --- | --- | --- | --- | --- |
| miRNA cluster^*^ | Abundance (rank) | Test score (d) | Fold-change | Q-value (%) |
| cluster-hsa-mir-210(1) | 77 | 103 | 6.8 | 0 |
| cluster-hsa-mir-331(1)^†^ | 113 | 83 | NA | 8.615 |
| cluster-hsa-mir-370(1)^†^ | 100 | 72 | NA | 8.615 |
| cluster-hsa-mir-130b(2) | 91 | 71 | 4.2 | 8.615 |
| cluster-hsa-mir-423(2) | 35 | 69 | 2.1 | 8.615 |
| cluster-hsa-mir-203(1) | 80 | 67 | 4.8 | 8.615 |
| cluster-hsa-mir-223(1) | 22 | 65 | 2.3 | 8.615 |
| cluster-hsa-mir-383(1)^†^ | 90 | 65 | NA | 8.615 |
| cluster-hsa-mir-126(1) | 27 | 64 | 2.6 | 8.615 |
| cluster-hsa-mir-25(3) | 14 | 63 | 2.4 | 8.615 |
| *, “Cluster” refers to a cistron from which individual miRNA precursors are co-transcribed. The numbers in parenthesis represent the total number of miRNA precursors included in the cluster. †, The fold changes for miR-331, miR-370 and miR-383 are unavailable due to very low read counts in the control group. | | | | |
